# Vertebral Artery Dissection and Cerebellar Stroke in a Young Patient Following Neck Manipulation

**DOI:** 10.14740/jmc5304

**Published:** 2026-05-20

**Authors:** Ella Lahart, Linda Akbarshahi, Jacob Tadros, Eric Evans

**Affiliations:** a GME Research Department, Northeast Georgia Medical Center, Gainesville, GA, USA; b Family Medicine, Northeast Georgia Medical Center, Gainesville, GA, USA

**Keywords:** Vertebral artery dissection, Cerebellar infarction, Patent foramen ovale

## Abstract

Vertebral artery dissection (VAD) is an uncommon but critical cause of ischemic stroke in young adults. While some cases of VAD are associated with trauma, many occur spontaneously or without clearly identifiable precipitating events. This case report describes a 33-year-old male with a history of migraines and attention deficit hyperactivity disorder (ADHD) who presented with worsening left-side headache, neck pain, nausea, and left arm paresthesia. His initial presentation to urgent care resulted in conservative treatment without improvement. The patient later presented to the emergency department following persistent symptoms and a recent history of chiropractic visits, based solely on patient self-report. Computed tomography angiography (CTA) revealed vertebral artery aneurysm and pseudoaneurysm, magnetic resonance imaging (MRI) demonstrated an acute lacunar infarct in the left cerebellar hemisphere, and magnetic resonance angiography (MRA) confirmed bilateral VAD. Transesophageal echocardiography (TEE) later revealed a patent foramen ovale (PFO), though no immediate intervention was required. The patient was managed with dual antiplatelet therapy and supportive medications for migraine and vertigo. This case warrants reporting due to the patient’s atypical age, absence of clear trauma, and the diagnostic delay caused by overlapping migraine symptoms. While a temporal association with recent neck manipulation was noted, causation cannot be established. Early recognition and appropriate imaging are essential to prevent disabling outcomes and guide proper management in at-risk populations.

## Introduction

Strokes are the second leading cause of death worldwide and in the United States, with an estimated 20% of people that will encounter one in their lifetime in high-income countries [1, 2]. A subtype of strokes that are relevant in this context is lacunar stroke, which results when small blood arteries within the brain become blocked, leading to areas such as the thalamus or basal ganglia to be affected [3]. While most strokes occur in older adults, and lacunar strokes have known risk factors like hypertension, diabetes, and unhealthy lifestyle, a significant proportion of ischemic strokes in young adults are attributed to cervical artery dissection (CeAD), including vertebral artery dissection (VAD) [4]. The vertebral arteries originate from the subclavian artery in the thorax, supplying blood flow to the cerebellum, brainstem, and anterior spinal cord [4]. Patients may initially present with nonspecific symptoms such as headache, neck pain, or dizziness [5].

In clinical practice, a suspected VAD is typically determined using computed tomography angiography (CTA) or magnetic resonance angiography (MRA) [4]. Additionally, magnetic resonance imaging (MRI) of the brain may reveal acute infarcts [4]. A transesophageal echocardiography (TEE) is often used to evaluate for potential cardiac embolic sources, such as patent foramen ovale (PFO), which is a known contributor to strokes in younger patients [6]. A PFO is a small hole between the two upper chambers of the heart, specifically between the right and left atrium [6].

Here, we present a case of a 33-year-old male with a history of migraine and attention deficit hyperactivity disorder (ADHD) who was ultimately diagnosed with VAD and CeAD following an initially misleading clinical course. The patient also reported multiple chiropractic visits, based solely on patient self-report, prior to diagnosis. This report underscores the importance of early vascular imaging in young adults with atypical migraine features, especially when accompanied by focal neurological signs. This case is atypical due to the fact that VAD is typically present in individuals aged between 40 and 60 years old, often following trauma, making this patient’s presentation atypical [4].

## Case Report

A 33-year-old male with a known history of migraines and ADHD presented to urgent care in early 2025. Additionally, it was noted that the patient has a family history of migraines and early-onset heart disease; his father had a myocardial infarction in his 40s and a cerebrovascular accident in his mid-70s. The patient initially reported symptoms of pain on the left side of the neck, face, and head; a headache localized to the left side with increased sensitivity to light and sound; nausea; vomiting; and paresthesia in the left arm and fingers. He was prescribed cyclobenzaprine at urgent care. However, after 2 weeks without improvement and multiple chiropractor visits (based solely on patient self-report), the patient presented to the emergency department. On initial emergency department presentation, neurological examination revealed left upper extremity paresthesia without motor weakness.

A computed tomography (CT) scan of the brain was ordered, which showed no acute hemorrhage or mass effect. A CTA of the neck and head was subsequently performed and revealed findings concerning vertebral artery aneurysm and pseudoaneurysm. MRI of the brain showed an acute lacunar infarct in the left cerebellar hemisphere. An MRA of the neck was then performed to confirm a bilateral VAD. The patient was prescribed aspirin and Plavix for stroke prevention. He was advised to follow up with cardiology and was referred to physical therapy for neck and vertigo management. A TEE was ordered by cardiology 2 months after initial symptoms started and revealed a PFO. The patient was advised to continue the current medications and to follow up with neurology and vascular surgery. During this follow-up, a cerebellar stroke secondary to VAD was confirmed. Based on these findings, there was no concern for immediate PFO closure, as the opening was small and unlikely to be contributing to the patient’s symptoms.

Currently, 3 months after the initial onset of symptoms, the patient is managing his final diagnosis of VAD and an acute lacunar infarct. His prescribed medications included 81 mg of aspirin daily, 75 mg of Plavix daily, and a standard dose of atorvastatin for stroke prevention; 60 mg of Qulipta daily for migraine management; 50 mg of Lyrica twice daily for chronic pain related to the VAD; and 25 mg of Meclizine at bedtime for vertigo. He was also referred to physical therapy for vertigo and neck pain, and he was scheduled for follow-ups with vascular surgery for the VAD and with neurology in 12 weeks for stroke and headache management.

## Discussion

Although relatively uncommon, VAD is an important cause of ischemic stroke in young adults, occurring at a rate of 1–1.1 per 100,000 individuals annually [7]. While commonly associated with trauma, spontaneous cases like the one described here can present diagnostic challenges due to nonspecific symptoms and atypical patient demographics [8]. The patient, a 33-year-old male with a history of migraines contributed to initial diagnostic uncertainty, as migraine symptoms may overlap with early manifestations of VAD [8].

The patient also reported multiple chiropractic visits, based solely on patient self-report, prior to diagnosis. The temporal proximity of these visits to symptoms progression was noted; however, it cannot establish causation at this time. Several case-control studies and systemic reviews suggest that patients with evolving dissection may seek care for neck pain or headache before diagnosis, creating a temporal association rather than a causal one.

This case illustrates the importance of timely vascular imaging in evaluating persistent or atypical headache and neck pain symptoms. As shown in Figure 1, the axial CTA images of the head and neck revealed changes suspiciously for vertebral artery aneurysm or pseudoaneurysm (Fig. 1a, b). These findings prompted further imaging with MRI and MRA, ultimately confirming a bilateral VAD. Notably, Figure 2 provides sagittal MRI evidence of cervical spine pathology, including moderate neural foraminal narrowing and possible central cord signal abnormality, additional findings that may have contributed to the patient’s symptoms and increased vulnerability to dissection (Fig. 2).

**Figure 1:**
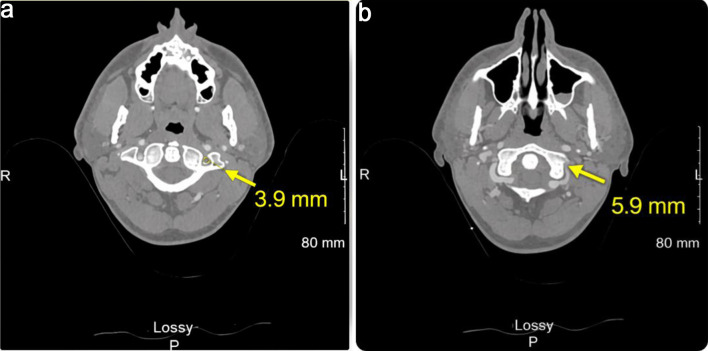
Axial computed tomography angiography image of the head and neck acquired in early 2025 (series 2, image 156 (a) and image 162 (b); slice thickness: 1.25 mm; location: –58.25 mm). Impressions indicate acute lacunar infarction in the left cerebellar hemisphere and small venous angioma in the left parietal lobe.

**Figure 2:**
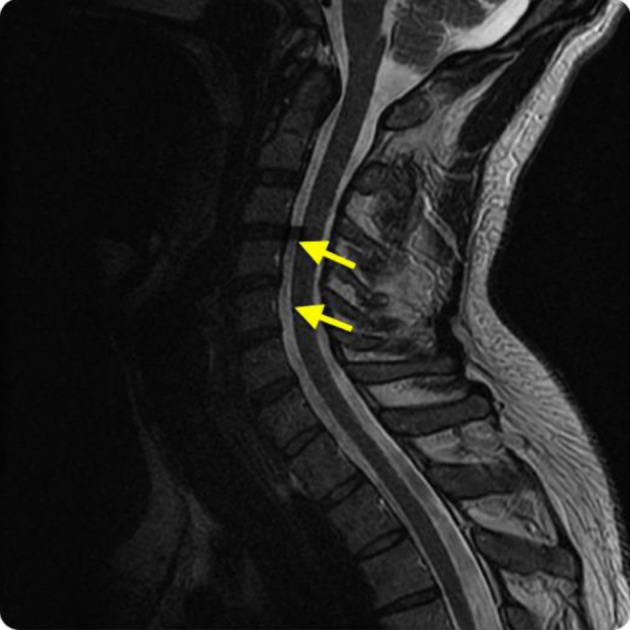
Sagittal magnetic resonance angiography of cervical spine without contrast acquired early to mid-2025 (image 11, slice thickness: 3.0 mm; location: 15.96). Impressions indicate moderate neural foraminal narrowing at C3-C4 and C4-C5 with possible central punctate T2 signal abnormality within the central cord at C6-C7.

Given the patient’s age and lack of recent trauma, features that are not typical for VAD, this case demonstrates the importance of considering this diagnosis in young patients presenting with unexplained neurological symptoms. In a similar case involving a young patient, a 41-year-old male presenting with severe headaches, neck pain, and dizziness was later diagnosed with VAD following a delayed ischemic stroke [9]. Similar to this case, there was no recent trauma, which made it difficult to diagnose VAD [9]. Unlike in this case however, the patient did not have an extensive medical history, like family history of early on-set heart disease, migraines, and multiple chiropractic visits. Migraines are not only a common risk factor for VAD, but also affect individuals’ risk of developing CeAD [10]. In one study it was found that individuals with migraines, particularly those without aura, have a 1.74 higher risk ratio compared to the general population [10]. This emphasizes the importance of maintaining awareness in patients with migraines presenting with new, atypical, or persistent neurological symptoms [10]. During the diagnostic and treatment process, this case brought light to the patient discovering that he has PFO. However, it was later determined that it did not need to be treated currently.

VAD can often mimic other conditions, which can lead to diagnostic delays or misdiagnoses [2]. In this case, the patient was initially treated after undergoing chiropractic neck manipulation, which occurred in temporal proximity to symptom progression; however, no causal relationship can be established. Early imaging, such as CTA or MRA, is crucial in identifying VAD and differentiating it from other potential causes of stroke [2]. This approach can facilitate timely intervention and improve patient outcomes [2].

The management of VAD involves anticoagulation therapy to prevent thromboembolic events, as well as symptomatic treatment for associated conditions such as migraines and vertigo [4]. In this case, the patient was prescribed aspirin, Plavix, and statins for stroke prevention, along with medications for migraine and vertigo management [4]. Additionally, the patient was advised to avoid neck manipulation, which could have exacerbated the risk of VAD [4]. Preventive measures, including the use of migraine prophylactic medications like beta-blockers, may reduce the risk of VAD in susceptible individuals [11].

This case highlights the importance of educating healthcare providers about the potential for VAD in young adults, particularly those with a history of migraines [4]. Clinicians should be aware of the signs and symptoms of VAD and consider it in the differential diagnosis when evaluating patients with unexplained neurological symptoms [4]. The presentation of VAD in a young, healthy individual without a history of trauma underscores the need for heightened awareness of this condition in clinical practice [4]. Early recognition through appropriate imaging and prompt management are essential to prevent adverse outcomes [4]. This case also emphasizes the association between migraines and an increased risk of VAD, highlighting the importance of considering vascular causes in patients with a history of migraines presenting with new or atypical neurological symptoms [4]. Future research using retrospective and case-control methodologies may help clarify risk factors and associations in CeAD. Additionally, the long-term significance of incidental PFO findings in spontaneous VAD cases remains an important area for investigation [4]. By maintaining a high index of suspicion and employing early diagnostic strategies, healthcare providers can improve the detection and management of VAD in young adults, ultimately enhancing patient outcomes [4].

### Conclusion

This case highlights VAD as an important cause of stroke in young patients presenting with persistent or atypical neurological symptoms. Diagnostic delay may occur due to overlap with migraine features. Early vascular imaging is essential for accurate diagnosis. While the patient reported recent chiropractic visits, based solely on patient self-report, this represents a temporal association only, and causation cannot be established.

### Learning points

VAD should be considered in young patients presenting with atypical headaches and neurological symptoms, as early recognition is essential for appropriate management. Timely vascular imaging, including CTA or MRA, plays a critical role in establishing the diagnosis. The presence of migraine may further complicate the clinical picture due to overlapping symptoms, potentially contributing to diagnostic delay. Additionally, clinical histories, including prior interventions, are often based on patient self-report and may be subject to recall bias. Finally, associations identified in case reports should be interpreted with caution, as such studies are not designed to establish causation.

## Data Availability

All data relevant to this case report are included within the article. Additional information may be made available upon reasonable request.

## Abbreviations

ADHD: attention deficit hyperactivity disorder
CeAD: cervical artery dissection
CT: computed tomography
CTA: computed tomography angiography
MRA: magnetic resonance angiography
MRI: magnetic resonance imaging
PFO: patent foramen ovale
TEE: transesophageal echocardiography
VAD: vertebral artery dissection
